# Fabrication of polyamide thin film composite membranes using aliphatic tetra-amines and terephthaloyl chloride crosslinker for organic solvent nanofiltration

**DOI:** 10.1038/s41598-023-38269-5

**Published:** 2023-07-20

**Authors:** Abdul Waheed, Umair Baig, Isam H. Aljundi

**Affiliations:** 1 Interdisciplinary Research Center for Membranes and Water Security, King Fahd University of Petroleum and Minerals, 31261 Dhahran, Saudi Arabia; 2 Chemical Engineering Department, King Fahd University of Petroleum & Minerals (KFUPM), 31261 Dhahran, Saudi Arabia

**Keywords:** Materials science, Chemistry, Chemical engineering, Organic chemistry, Polymer chemistry, Surface chemistry

## Abstract

Given the huge significance of organic solvents in several industrial processes, the use of membranes for recovering the solvents has evolved into an industrially viable process. The current work has been focused on studying the effect of minor changes in the chemistry of the reacting monomers on the organic solvent nanofiltration/solvent resistance nanofiltration (OSN/SRNF) performance of the membranes. The two aliphatic amines with varying aliphatic chain lengths between primary and secondary amines were selected for this purpose. Based on the structure of the resultant active layer, the Janus nanofiltration performance of the membrane was evaluated. The two membranes, 4A-TPC@crosslinked PAN and 4A-3P@crosslinked PAN were fabricated by using two different tetra-amines, 4A (*N*,*N*′-bis(3-aminopropyl)ethylenediamine) and 4A-3P (*N*,*N*′-Bis(2-aminoethyl)-1,3-propanediamine) crosslinked with terephthaloyl chloride (TPC) on a crosslinked polyacryonitrile (PAN) support through interfacial polymerization (IP). The presence of multiple hydrophobic –CH_2_– groups in the structures of the aliphatic amines 4A and 4A-3P develops hydrophobic sites in the hydrophilic polyamide active layers of the membranes. In addition, 4A has two secondary amino groups separated by ethylene (–CH_2_–CH_2_–) groups, whereas in 4A-3P, the two secondary amino groups are separated by propylene (–CH_2_–CH_2_–CH_2_–) leading to variation in the structural features and performance of the two membranes. Both membranes were fully characterized by several membrane characterization techniques and applied for OSN/SRNF using both polar (methanol, ethanol, and isopropanol) and non-polar (*n*-hexane and toluene) solvents. Different dyes (Congo red, Eriochrome black T, and Methylene blue) were used as model solutes during the filtration experiment. The 4A-3P-TPC@crosslinked PAN showed *n*-hexane and toluene flux of 109.9 LMH and 95.5 LMH, respectively. The Congo red (CR) showed the highest rejection, reaching 99.1% for the 4A-TPC@Crosslinked PAN membrane and 98.8% for the 4A-3P-TPC@Crosslinked PAN membrane.

## Introduction

Organic solvents are of prime significance in several industrial processes, ranging from pharmaceuticals to petrochemicals. The reuse of highly precious organic solvents is of utmost economic significance as it can lower the demand for fresh organic solvents^1^. Hence, the chemical industries are constantly improving their processes to (i) meet the stringent environmental regulations and (ii) increase their profit^2^. Among the chemical industries, the pharmaceutical industries are heavily dependent on the consumption of organic solvents, where organic solvents account for 80–90% of the total mass in the process^3^. The most common solvent wastes generated by pharmaceutical companies include methanol, dichloromethane, toluene, acetonitrile, and chloroform. Among the different organic solvent wastes, methanol waste has been estimated to reach 44.8 × 10^6^ kg per year. Compared to conventional processes such as distillation and adsorption, membrane-based separations are less energy intensive, eco-friendly, have a footprint, cost-effective for product isolation and concentration^4^.

With the development of thin film composite (TFC) membranes, several separation processes have been optimized^5–7^, which is due to certain salient features of the TFC membranes such as chemical and physical stability, adaptability to different feed compositions, and easy availability with a wide range of pore sizes for a desired application^8–10^. TFC membranes consist of a thin selective layer deposited on top of a porous polymeric ultrafiltration support. Generally, the porous polymeric ultrafiltration support is prepared through wet phase inversion by using water as a non-solvent in the process. On the other hand, the active layer is grown on the ultrafiltration support through interfacial polymerization (IP)^11^. Given the versatility of the IP process, a huge variety of TFC membranes have been fabricated in the literature by either altering the chemistry of reacting monomers or changing the other parameters of IP^12^.

The variations in the chemistry of the active layers of TFC membranes have led to remarkable applications. Solomon et al. reported a polyamide (PA) TFC membrane by using conventional meta-phenylenediamine (MPD) as an aqueous monomer and trimesoyl chloride (TMC) as a non-aqueous monomer during IP. The membrane was prepared on a polyimide (PI) substrate and showed solvent resistance^13^. Another group reported the green synthesis of a PA TFC membrane by using polyethylenimine (PEI) as an amine solution cross-linked with TMC on porous PAN support. The IP reaction was carried out using decanoic acid as the organic phase. The membrane was able to reject the majority of the tested dyes with a molecular weight cut-off (MWCO) of 650 g mol^−1^^14^. Recently, molecule-based design strategies have been explored by the research community for manipulating the pore structure and physicochemical properties of organic solvent nanofiltration/solvent-resistant nanofiltration (OSN/SRNF)^15^ membranes. In a recent study by Alduraiei et al., fluorine-containing diamine, 5-trifluoromethyl-1,3-phynelenediamine (TFMPD), was crosslinked with TMC and 4,4′-(hexafluoroisopropylidene)bis(benzoyl chloride) (HFBC) during IP. Since the presence of fluorine atoms in the active layer gives a hydrophobic feature to the membrane, the resulting membranes were used for cleaning non-polar solvents. The TFMPD-HFBC membrane showed a toluene permeance of 10 L m^−2^ h^−1^ bar^−1^^16^. Thijs et al. used a novel approach to tune the chemistry of the active layer of the TFC membrane by growing an active layer consisting of polymers of intrinsic microporosity (PIMs). In their work, several amine monomers with 2.5–4 amines per molecule were crosslinked with binaphthalene-based di(acid chloride) through IP on Matrimid support. The fabricated membrane showed an increase in acetonitrile permeance by a factor of 20 compared to a conventional MPD/TMC membrane. By comparing the structure and performance of the different fabricated membranes, the team found that changing the number of amine groups per molecule and altering the length of alkyl chains on binaphthalene-based di(acid chloride) altered the performance of the membranes^17^. In one such instance, Liu et al. constructed Janus pathways by using cyclodextrin (CD) in the membrane. The presence of CD in the constructed OSN/SRNF membrane allowed passage of both polar and non-polar solvents, which is attributed to the inner hydrophobic cavities and outer hydrophilic spaces of CD^18^. However, the larger pore size of CD lowers the rejection of the membrane. Similarly, Li et al. developed a Janus membrane by using β-cyclodextrin (BCD) as a monomer during IP on a polyelectrolytes-based interlayer developed through layer-by-layer self-assembly. The membrane was able to permeate both polar and non-polar solvents with permeances reaching 5.8 LMH/bar for methanol and 7.0 LMH/bar for hexane along with a 91.9% rejection of methyl orange^19^. In another work, Li et al. used *m*-xylylene diamine (*m*-XDA) as an aqueous monomer crosslinked with TMC leading to a Janus membrane for the passage of both polar and non-polar solvents. The addition of an additional methylene –CH_2_– group in *m*-XDA rendered the hydrophobic features to the hydrophilic polyamide active layer of the membrane^20^. Hence, minor variations at the molecular level of reacting monomers lead to different structural features targeting a specific application. Therefore, the need of the day is to come up with new membranes with structural, physical and chemical stability by tuning the chemistry of reacting monomers.

The current study was carried out by using the linear aliphatic amines 4A-3P and 4A on a crosslinked PAN support. The study was carried out through interfacial polymerization between either 4A-3P and TPC or 4A and TPC on crosslinked PAN support. In comparison to the previous studies where cyclic amines such as piperazine or aromatic amines such as meta-phenylenediamine (MPD) were used, we have used linear aliphatic amines 4A and 4A-3P crosslinked with an organic phase containing terephthaloyl chloride (TPC) as a cross-linker. The presence of multiple methylene –CH_2_– groups in the structure of tetra-amines (4A and 4A-3P) introduced several hydrophobic sites inside the hydrophilic polyamide active layer leading to the passage of both polar (methanol, ethanol, and isopropanol) and non-polar (*n*-hexane and toluene) solvents. Moreover, another variation in the chemistry of the newly developed polyamide active layer was also introduced as in the case of 4A both primary (–NH_2_) and secondary (–NH) amines were separated by ethylene –CH_2_–CH_2_– groups. In the case of 4A-3P, the two primary (–NH_2_) amino groups are separated by ethylene –CH_2_–CH_2_– and the two secondary amino (–NH) groups are separated by propylene –CH_2_–CH_2_–CH_2_– chain. This variation at the molecular level could potentially lead to different morphological and structural features leading to varied OSN performance. The fabricated membranes were extensively characterized by using a scanning electron microscope (SEM), attenuated total reflectance Fourier transform infrared (ATR-FTIR) spectroscopy, water contact angle (WCA), atomic force microscopy (AFM), energy dispersive X-ray (EDX) and elemental mapping. The fabricated membranes were used for OSN/SRNF applications by using both polar and non-polar solvents while different dyes were used as model solutes during filtration experiments.

## Materials and methods

### Materials

Terephthaloyl chloride (TPC; ≥ 99%), *N*,*N*′-Bis(2-aminoethyl)-1,3-propanediamine (4A-3P Amine; 97%), *N*,*N*′-bis(3-aminopropyl)ethylenediamine (4A; 90%), Triethylamine (TEA; ≥ 99.5%), Polyacrylonitrile (PAN; ≥ 99%; average molecular weight = 150,000), *n*-Hexane (≥ 99%), *N*,*N*′-dimethyl formamide (DMF, ≥ 99%), Polyethylene Terephthalate (PET) fabric, Methylene blue (MB; ≥ 99%), Eriochrome black T (EBT; ≥ 99%), and Congo red (CR; ≥ 99%) were all purchased from Sigma (St. Louis, USA). Deionized (DI) water from the in-lab setup was used for all the experiments.

### Fabrication of PAN support and crosslinking of PAN support

The PAN dope solution was prepared by dissolving 12 g of perfectly dried PAN in 88 g of DMF. The solution was stirred at room temperature overnight, leading to a transparent solution of PAN. The PAN dope solution was allowed to stand until all of the air bubbles were completely removed. The PAN support was prepared by spreading an appropriate amount of PAN dope solution on a PET sheet affixed to a glass plate. Then the PAN was cast by using a custom-made Doctor’s blade (100 µm slit width) on PET support. Immediately after casting PAN, the support was dipped in a deionized water bath and allowed to stand, leading to phase inversion. Hence, PAN/PET ultrafiltration support was fabricated. The PAN support was crosslinked by dipping an appropriate piece of PAN/PET support in a 25% (v/v) hydrazine (NH_2_–NH_2_) aqueous solution at 70 °C for 6 h. Then the crosslinked PAN support was thoroughly washed with an excess of DI water.

### Fabrication of membranes through interfacial polymerization

**Figure 1:**
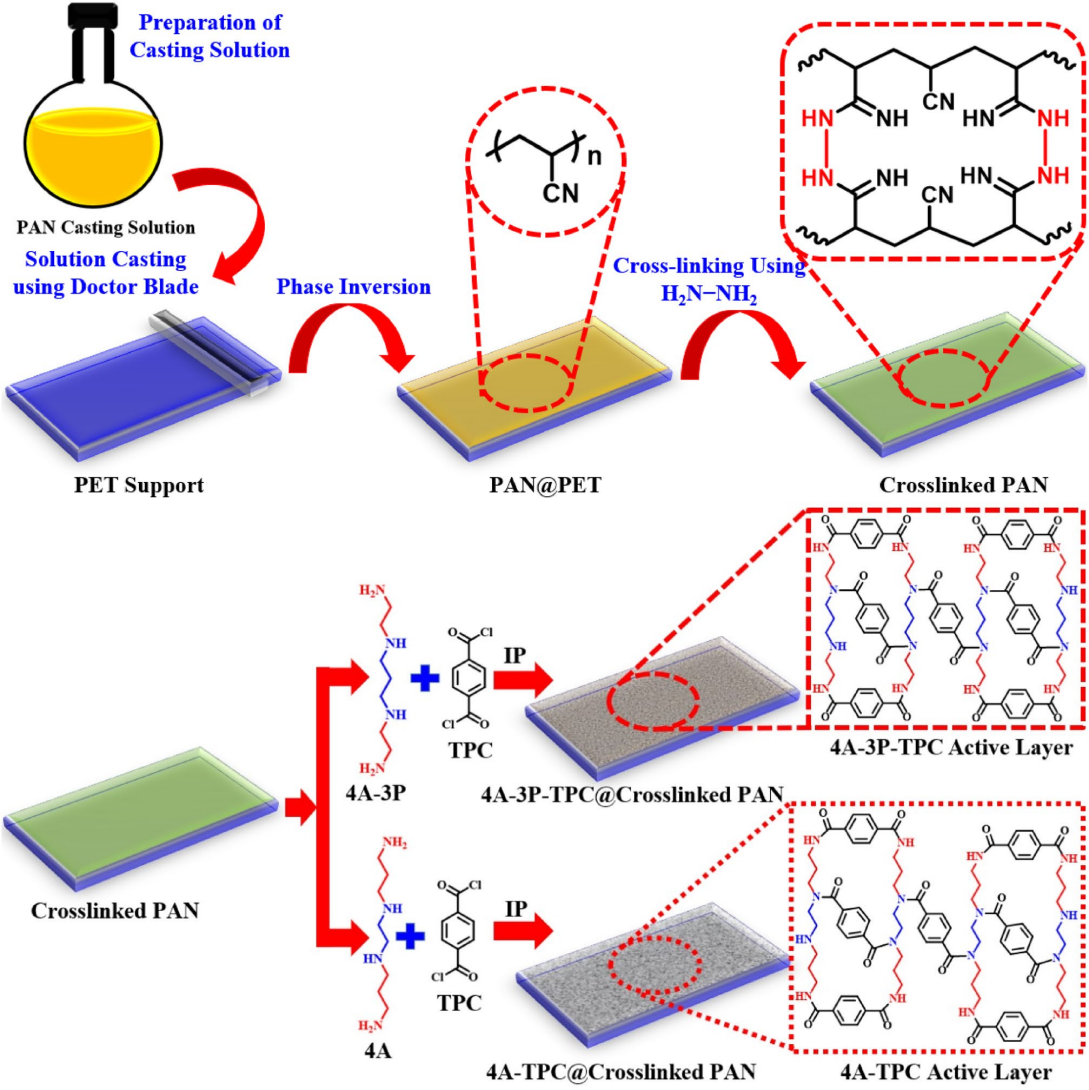
Different stages of fabrication of crosslinked PAN, 4A-TPC@crosslinked PAN, and 4A-3P-TPC@crosslinked PAN membranes.

The membranes were fabricated through an IP reaction on crosslinked PAN support. The crosslinked PAN support was dipped in an aqueous solution of either 4A-3P or 4A amine for 10 min with continuous shaking on a seesaw shaker. The amine-impregnated crosslinked PAN support was taken out of the aqueous amine solution and excess amine was removed by using a rubber roller. Then amine impregnated crosslinked PAN support was dipped in a 0.15% (w/v) *n*-hexane solution of TPC for 60 s. Then the membrane was removed from the TPC solution and washed with fresh *n*-hexane to remove unreacted TPC from the membrane surface. Various steps adopted during the fabrication of both membranes are given in Fig. 1.

## Results and discussion

### Characterization of fabricated membranes

**Figure 2:**
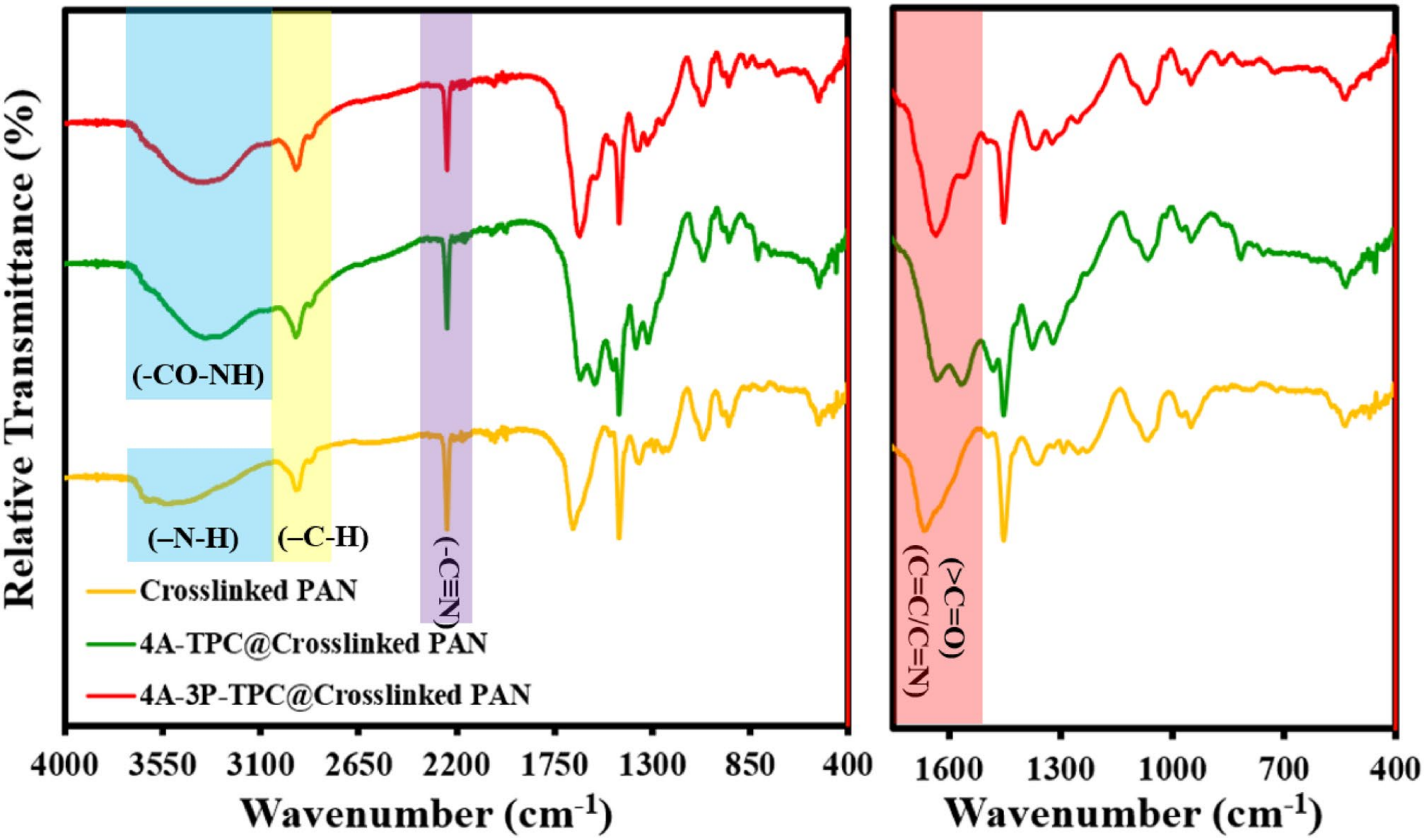
ATR-FTIR spectra of crosslinked PAN, 4A-TPC@Crosslinked PAN and 4A-3P-TPC@Crosslinked PAN membranes.

To establish and understand the structure of the different membranes, namely crosslinked PAN, 4A-TPC@crosslinked PAN, and 4A-3P-TPC@crosslinked PAN were characterized by ATR-FTIR. ATR-FTIR of all of the membranes is given in Fig. 2. All of the anticipated functional groups have been identified in different membranes. Since all of the FTIR spectra of different membranes contain a broad peak in a range of 3600–3200 cm^−1^, this can be attributed to the stretching vibration of the N–H bond of the amide linkage (–CO–NH) of the polyamide active layer of 4A-TPC@Crosslinked PAN and 4A-3P-TPC@crosslinked PAN. Similarly, in the case of crosslinked PAN, the 3600–3200 cm^−1^ broad peak can be attributed to the existence of the –N–H bond generated during crosslinking using hydrazine (NH_2_–NH_2_) as a crosslinking agent. As we move further, there is a short but relatively sharp peak located at 2900–2800 cm^−1^ in all of the FTIR spectra of all membranes including crosslinked PAN. This characteristic peak is due to –C–H stretching of the aliphatic backbones of the polyamide active layer due to constituent amines 4A, 4A-3P and PAN support. Furthermore, a very sharp and deep peak was visible at around 2200 cm^−1^ which is due to residual nitrile (–C≡N) groups in PAN. A relatively medium peak of crosslinked PAN can be seen in the region of 1700–1650 cm^−1^ which can be attributed to the stretching vibration of C=C/C=N bonds that originated during the crosslinking event of PAN with hydrazine. However, in the case of 4A-TPC@crosslinked PAN and 4A-3P-TPC@crosslinked PAN membranes, a doublet peak is shown in the region of 1700–1600 cm^−1^. In addition to C=C/C=N bonds of PAN, 4A-TPC@Crosslinked PAN and 4A-3P-TPC@Crosslinked PAN membranes have carbonyl functional group (> C=O) of the primary/secondary amide linkages of polyamide active layers. The doublet in the region of 1700–1600 cm^−1^ is attributed to the C=C/C=N and > C=O bonds of PAN and polyamide active layers. Therefore, the ATR-FTIR spectra of crosslinked PAN, 4A-TPC@Crosslinked PAN and 4A-3P-TPC@Crosslinked PAN membranes have confirmed the presence of all the functional groups and bonds in their structure (Fig. 1).

**Figure 3:**
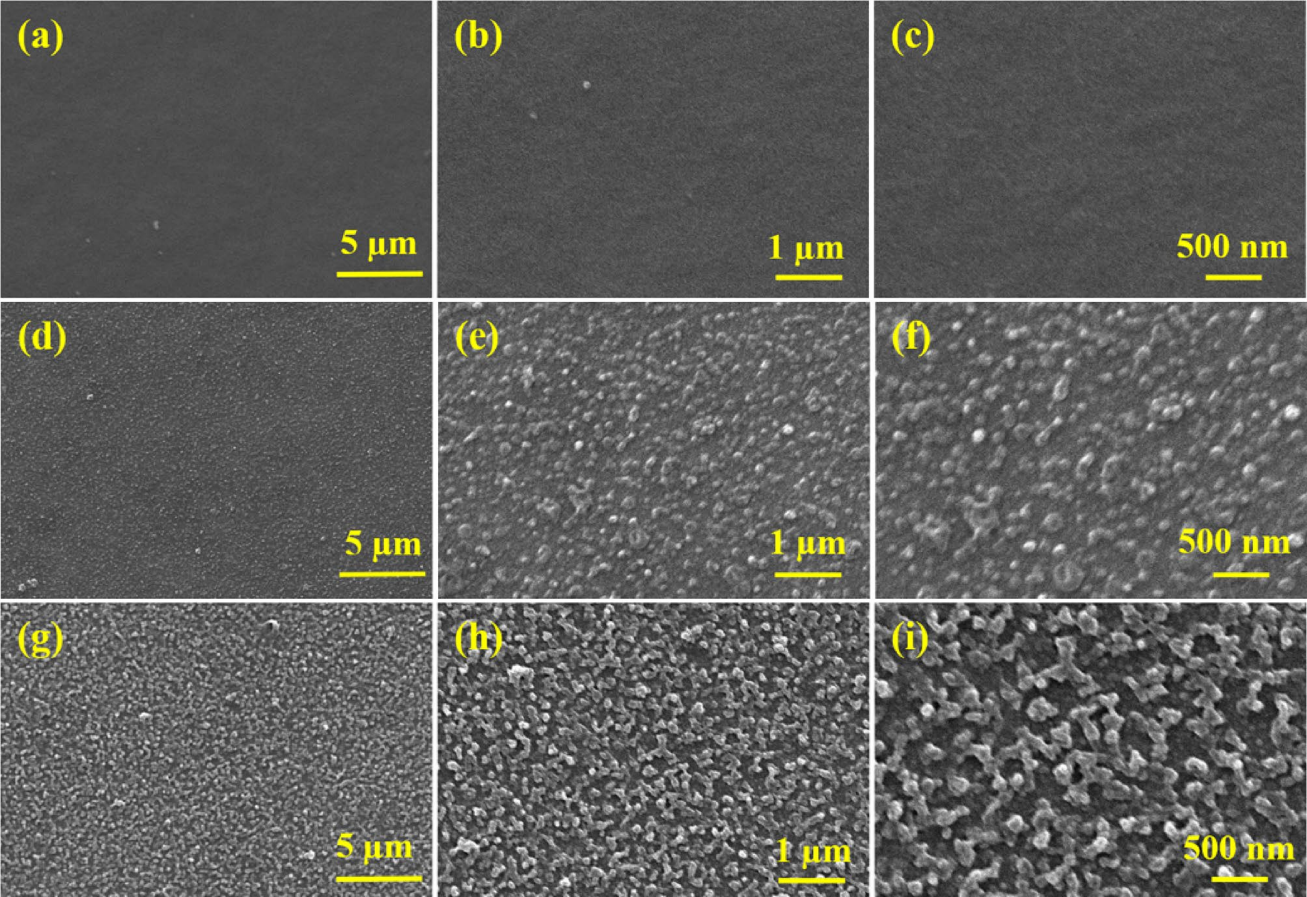
SEM micrographs of (**a**–**c**) Crosslinked PAN support, (**d**–**f**) 4A-TPC@Crosslinked PAN membrane and (**g**–**h**) 4A-3P-TPC@Crosslinked PAN membrane at different magnifications.

The membrane’s surface morphology was investigated by studying and analyzing the SEM micrographs of the crosslinked PAN support, 4A-TPC@Crosslinked PAN and 4A-3P-TPC@Crosslinked PAN membranes. The crosslinked PAN support appeared highly porous with a uniform sponge-like structure (Fig. 3a–c). After IP, the membrane surface appeared considerably rough owing to the growth of a polyamide active layer on support. In the case of 4A-TPC@Crosslinked PAN, the polyamide active layer appeared beaded with the beads embedded in the matrix of polyamide. The geometry of the 4A-TPC@Crosslinked PAN membrane resembles ridge and valley structure. However, the ridge and valley conformation is not perfectly adopted by 4A-TPC@Crosslinked PAN membrane as the valleys are filled with a polyamide network (Fig. 3d–f). In the case of 4A-3P-TPC@Crosslinked PAN membrane, the polyamide active layer perfectly matches the traditional ridge and valley configuration of the polyamide membranes (Fig. 3g–i). The variation in the pattern of growth of polyamide active layer on PAN support can be attributed to the change in structure of tetra-amine reacting during IP with the same crosslinker TPC. In the case of 4A-3P, secondary amine (–NH–) groups are located at the ends of propyl chain (3C atoms apart) while in the case of 4A, the secondary amine (–NH–) groups are separated by ethyl chain (2C atoms apart). The longer chain length provides a certain degree of freedom for penetration of TPC leading to extensive crosslinking and the formation of a special ridge and valley patterns as in the case of 4A-3P amine. In the case of 4A, the amine groups are comparatively close together offering less chance for TPC to penetrate. Hence, the 4A-3P-TPC@Crosslinked PAN membrane possesses a perfect geometry for efficient transfer of solvent molecules while rejecting the solutes as confirmed by the filtration experiments. Therefore, the chemistry of the reacting monomers is of utmost importance in tuning the performance of the nanofiltration membranes.

**Figure 4:**
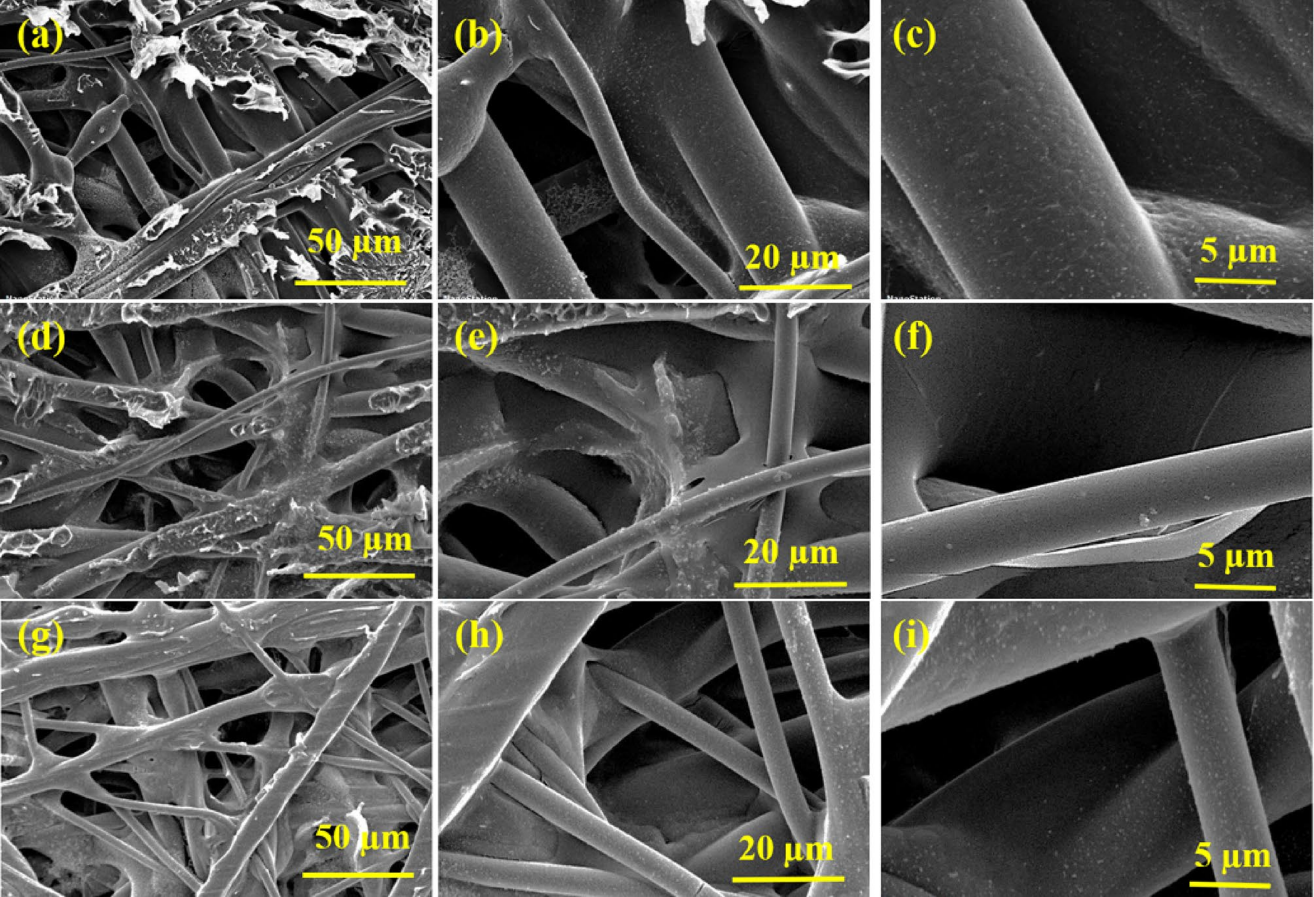
SEM micrographs of bottom surfaces of Crosslinked PAN support (**a**–**c**), 4A-TPC@Crosslinked PAN membrane (**d**–**f**) and 4A-3P-TPC@Crosslinked PAN membrane (**g**–**i**) at different magnifications.

Figure 4 shows the SEM micrographs of backsides of the PAN/PET support (Fig. 4a–c), 4A-TPC@Crosslinked PAN membrane (Fig. 4d–f) and 4A-3P-TPC@Crosslinked PAN membrane (Fig. 4g–i) at different magnifications. The morphological analysis of all the micrographs revealed that there was no growth of active layer on the backside of the (PET) membrane. This might be attributed to highly porous nature of the PET support which is not able to retain a considerable amount of amine monomer required for IP after dipping in a *n*-hexane solution of TPC. Hence, the highly porous nature of the support is desperately required for providing free passage to the solvents during filtration experiments.

**Figure 5:**
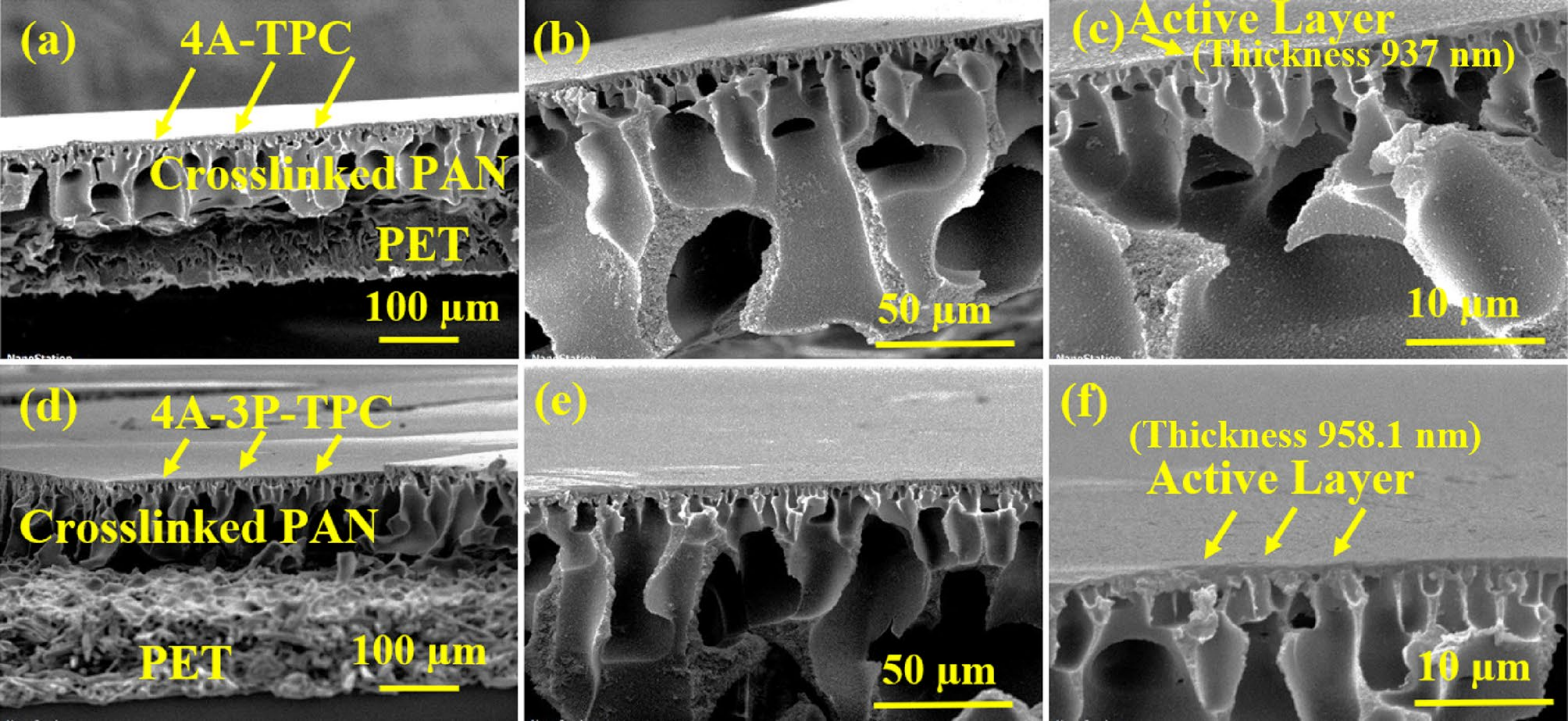
Cross-sectional SEM micrographs of 4A-TPC@Crosslinked PAN membrane (**a**–**c**) and 4A-3P-TPC@Crosslinked PAN membrane (**d**–**f**) at different magnifications.

The cross-sectional SEM micrographs of both membranes 4A-TPC@Crosslinked PAN (Fig. 5a–c) and 4A-3P-TPC@Crosslinked PAN (Fig. 5d–f) were recorded at different magnifications. In the case of both membranes, the cross-sections showed the presence of all three layers of TFC membrane which include highly porous and fibrous unwoven PET at the bottom of the membrane while the middle layer was the ultrafiltration PAN support showing uniformly distributed fingerlike projections and hence PAN/PET support provides an ideal support with minimum mass transfer resistance during filtration experiments. The third and most important separating layer was the highly dense and skin-like polyamide selective layer with a thickness of 937 nm for 4A-TPC@Crosslinked PAN and 958.1 nm for 4A-3P-TPC@Crosslinked PAN membranes. Hence, the cross-sectional SEM micrographs revealed a characteristically asymmetric TFC membrane structure which is the key to the separation capability of the TFC membranes.

**Figure 6:**
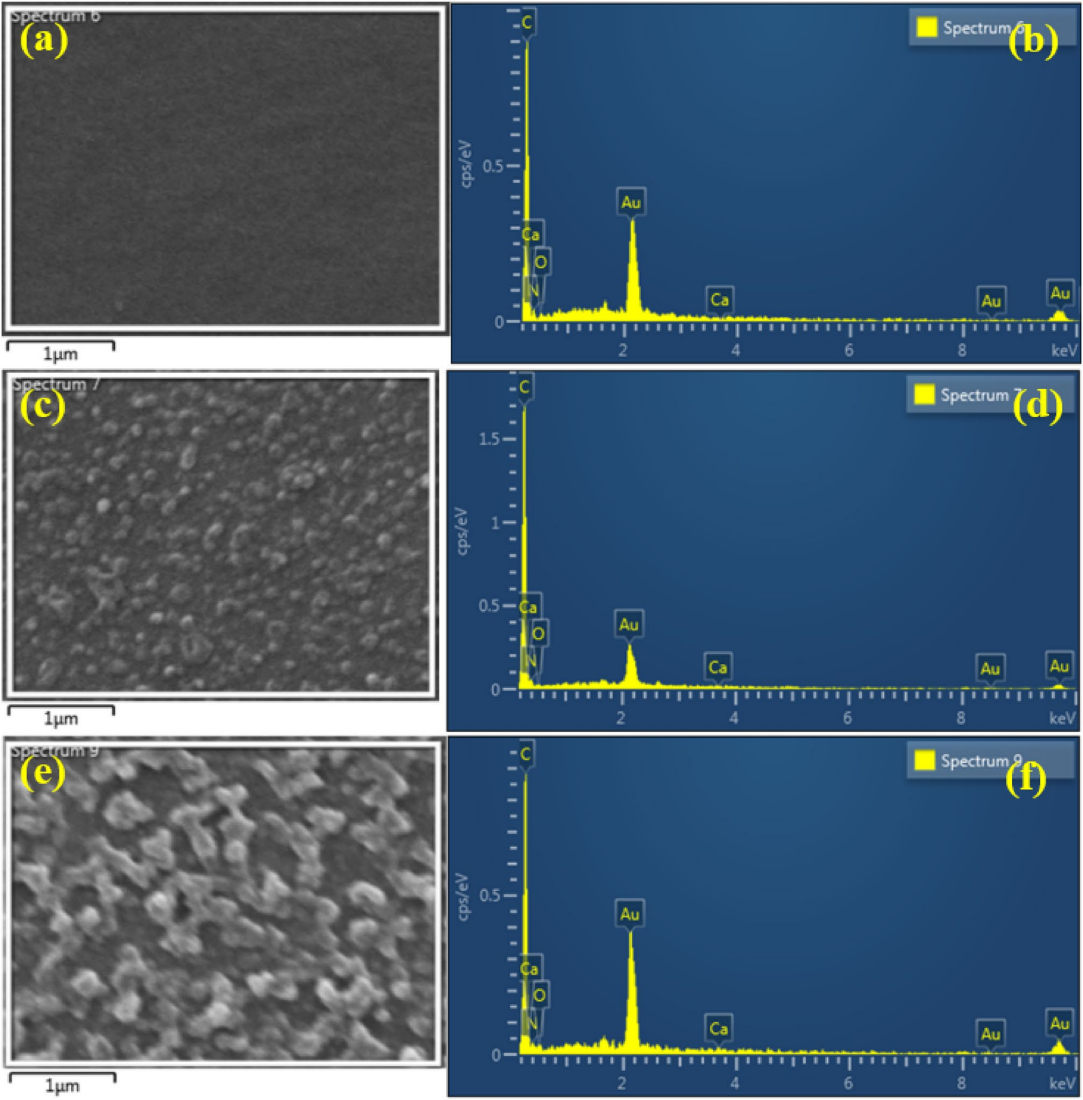
EDX analysis of (**a**–**b**) Crosslinked PAN support, (**c**–**d**) 4A-TPC@Crosslinked PAN membrane and (**e**–**f**) 4A-3P-TPC@Crosslinked PAN membrane.

The elemental composition of support, 4A-TPC@Crosslinked PAN membrane and 4A-3P-TPC@Crosslinked PAN membrane were estimated by energy dispersive X-ray (EDX) analysis as shown in Fig. 6. The EDX analysis of a selected area showed the presence of all of the elements that were present in contributing amines, TPC, PAN, and Hydrazine used during membrane fabrication.

**Figure 7:**
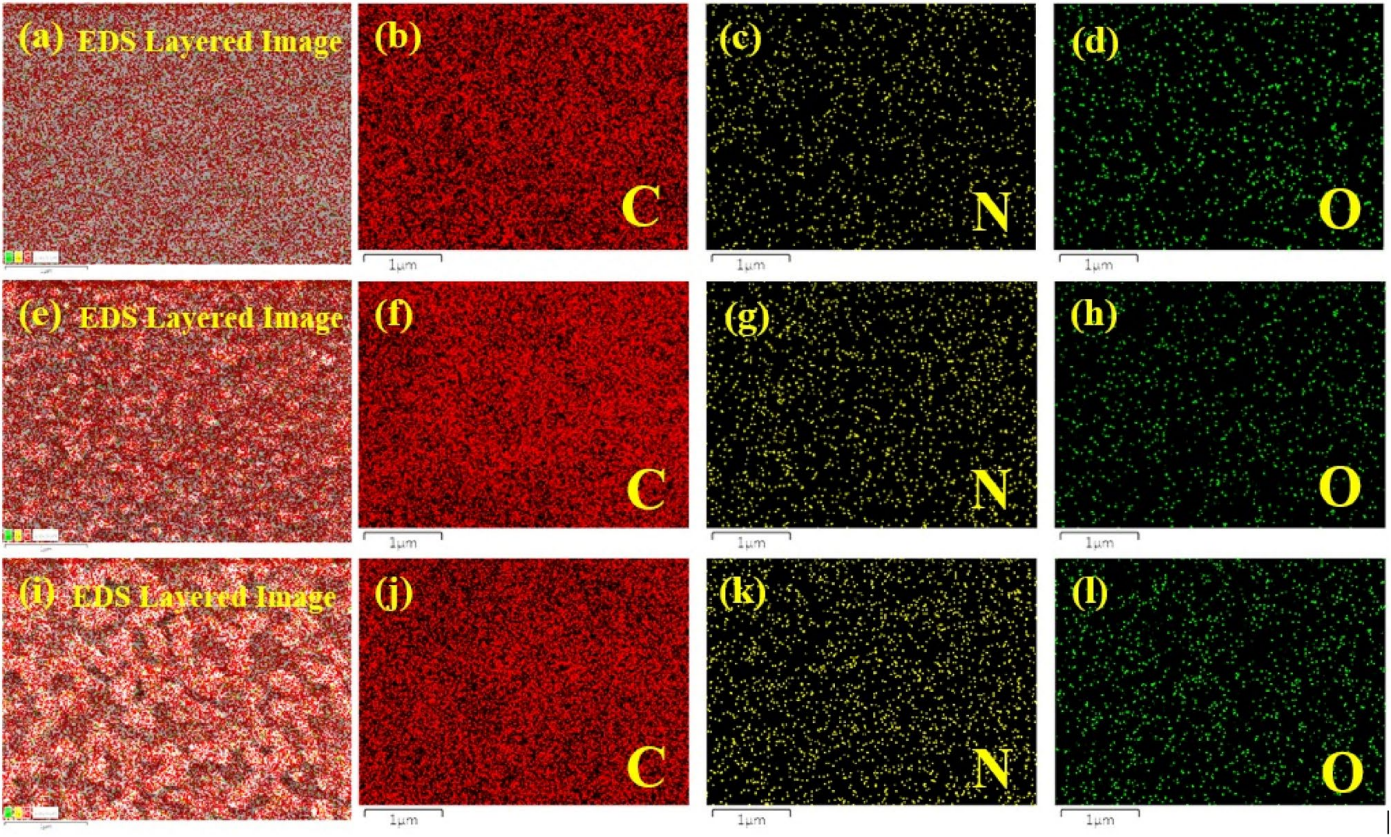
Elemental mapping of (**a**–**d**) Crosslinked PAN support, (**e**–**h**) 4A-TPC@Crosslinked PAN membrane and (**i**–**l**) 4A-3P-TPC@Crosslinked PAN membrane.

The elemental mapping analysis revealed a uniform distribution of all the elements throughout the entire membrane (Fig. 7).

**Scheme 1:**
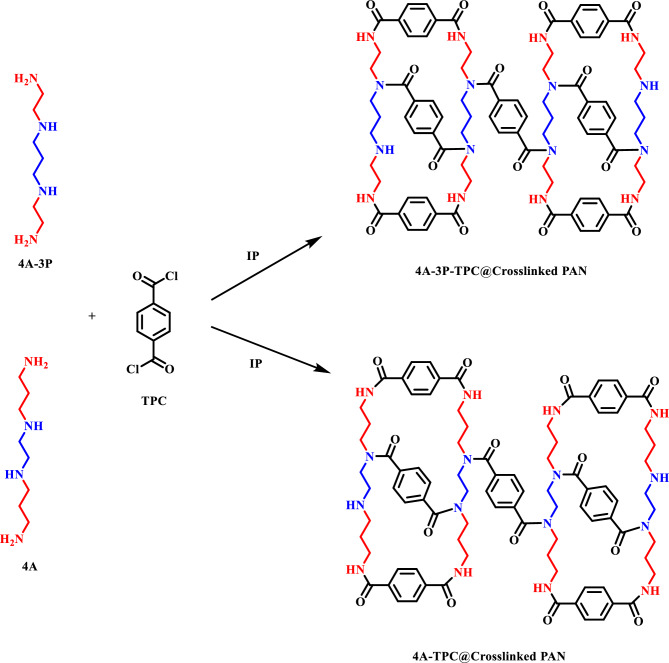
Proposed structure of active layers of 4A-TPC@Crosslinked PAN and 4A-3P-TPC@Crosslinked PAN.

The proposed structure of the active layers of the fabricated membranes is given in Scheme 1. The polyamide active layers are generated as a result of a reaction between tetra-amines (4A and 4A-3P) and terephthaloyl chloride (TPC). The 4A and 4A-3P amines have closely resembling structures with differences in the position of amino groups in the structure of tetra-amines. In case of 4A-3P amine, the two secondary amino groups (–NH–) are separated by propyl chain while the primary amino groups (–NH_2_) are ethyl chains. This particular structure of 4A-3P allows flexibility of binding of TPC with the neighbouring 4A-3P chains. However, in the case of 4A amine, the secondary amino groups (–NH–) are separated by ethyl chains and primary amino groups (–NH_2_) are separated by propyl chains. As the secondary amino groups are close together the incorporation of TPC in between is not flexible and uniform (Scheme 1). Therefore, the 4A-3P amine leads to the formation of a network with more uniform crosslinking in the polyamide active layers. The SEM analysis (Fig. 3g–i) confirmed the growth of more extensive crosslinking in the polyamide active layer of the membrane.

**Figure 8:**
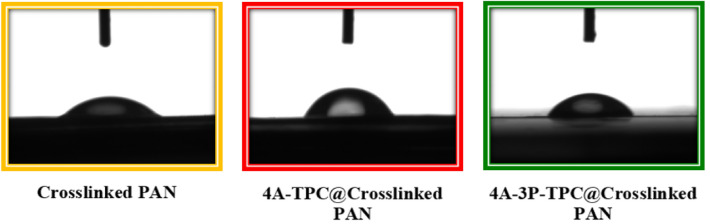
The water contact angles (WCAs) of Crosslinked PAN support, 4A-TPC@Crosslinked PAN membrane and the 4A-3P-TPC@Crosslinked PAN membrane.

The surface hydrophilicity of the support and fabricated membranes was determined by recording the water contact angle (WCA). It was observed that the WCA of the Hydrazine Crosslinked PAN support was found to be 45.9° and hence the crosslinked PAN support was considerably hydrophilic. The hydrophilicity of crosslinked PAN support can be attributed to the contribution of hydrazine introducing amino groups in the support. Furthermore, the hydrophilicity of crosslinked PAN can also be due to partial hydrolysis of the PAN support. However, after the growth of the polyamide active layer on crosslinked PAN support, the membrane surface hydrophilicity decreased as the WCA increased (65.1°) in the case of 4A-TPC@Crosslinked PAN membrane and 59.9° for 4A-3P-TPC@Crosslinked PAN (Fig. 8). This might be attributed to the covering of highly hydrophilic crosslinked PAN support. In addition, the inclusion of phenyl rings of TPC in polyamide active layer can also contribute to increasing the value of the WCA and decreasing the surface hydrophilicity of the membrane. Although the WCA of 4A-TPC@Crosslinked PAN and 4A-3P-TPC@Crosslinked PAN membranes were slightly increased, the values of WCAs are still considerably below 90°. Hence, both 4A-TPC@Crosslinked PAN and 4A-3P-TPC@Crosslinked PAN membranes are still quite hydrophilic. The WCA of a surface depends upon several factors of which surface roughness and chemical composition are of prime importance^21^. Generally, WCA decreases with a decrease in surface roughness increasing the hydrophilicity of the membrane or vice versa^22^. In the current study, compared to pristine crosslinked PAN support, the surface roughness of the fabricated membranes was increased. The average surface roughness was increased from 5.35 to 12.8 nm in the case of 4A-TPC@Crosslinked PAN while in the case of 4A-3P-TPC@Crosslinked PAN the roughness was increased to 28.7 nm. Therefore, the WCAs of 4A-TPC@Crosslinked PAN and 4A-3P-TPC@Crosslinked PAN were also increased in comparison to the Crosslinked PAN support. Among the fabricated membranes, 4A-3P-TPC@Crosslinked PAN has the highest surface roughness but the WCA of 4A-3P-TPC@Crosslinked PAN is slightly lower compared to 4A-TPC@Crosslinked PAN. Hence, in addition to the membrane surface roughness, surface chemistry also plays a role in controlling the hydrophilicity of the membranes. Generally, the presence of residual amino groups in the active layers of the membrane along with -COOH groups contributes to the hydrophilicity of the membranes^22, 23^.

**Figure 9:**
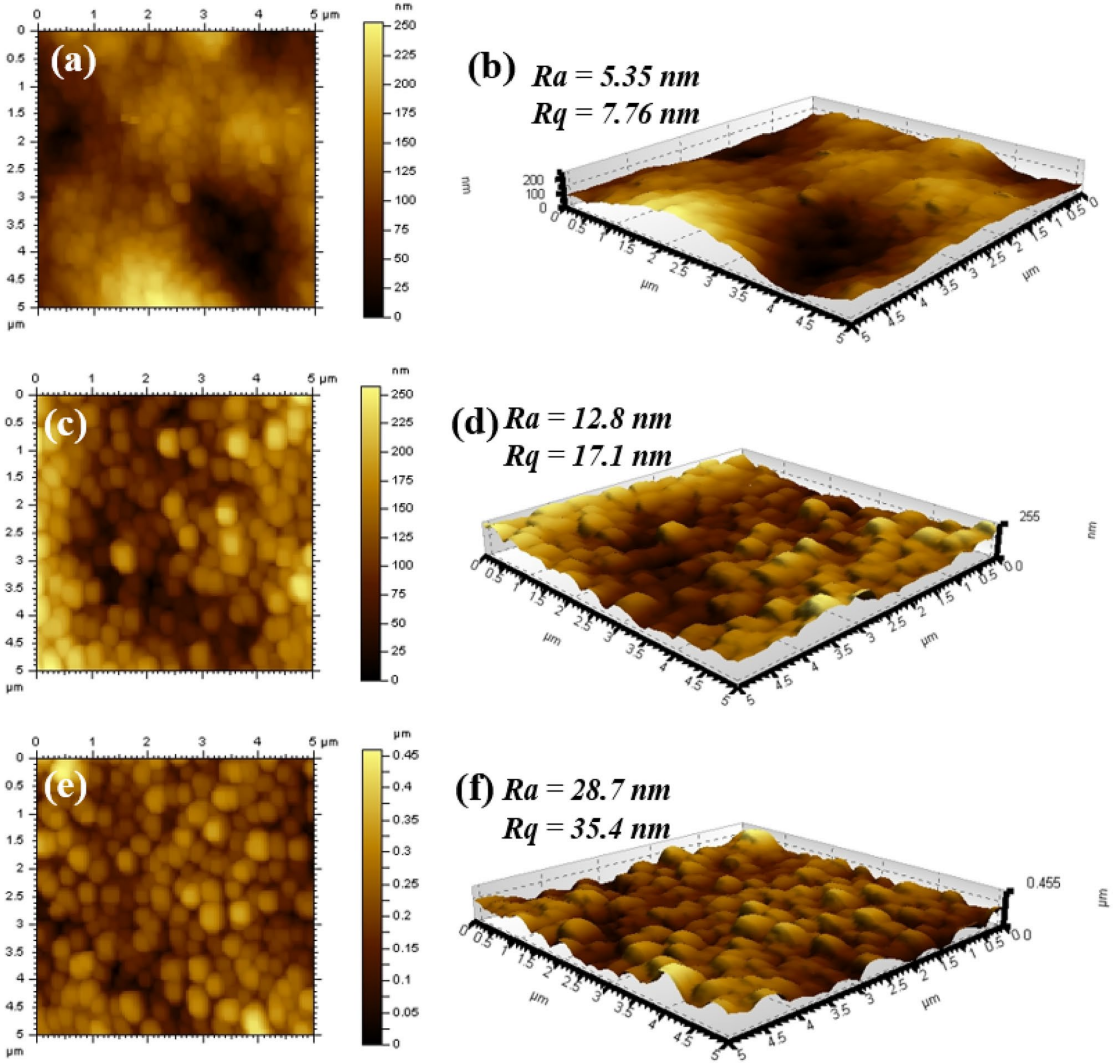
AFM images of (**a**,**b**) Crosslinked PAN support, (**c**,**d**) 4A-TPC@Crosslinked PAN membrane and (**e**,**f**) 4A-3P-TPC@Crosslinked PAN membrane.

Another salient feature of membranes is their surface roughness which was measured by recording atomic force microscopy (AFM) images of the crosslinked PAN, 4A-TPC@Crosslinked PAN membrane and 4A-3P-TPC@Crosslinked PAN membrane. The membrane average roughness (R_a_) and root mean square roughness (R_q_) were found to increase in the following order: crosslinked PAN > 4A-TPC@Crosslinked PAN membrane > 4A-3P-TPC@Crosslinked PAN membrane (Fig. 9). The R_a_ value (28.7 nm) of 4A-3P-TPC@Crosslinked PAN membrane was higher (12.8 nm) than 4A-TPC@Crosslinked PAN membrane. The higher R_a_ values in the case of 4A-3P-TPC@Crosslinked PAN membrane were attributed to the presence of large valleys and deep ridges as seen in Fig. 9e,f. The presence of ridge and valley conformation resembles commercial polyamide membranes and this conformation is ideal for the rejection of solutes and permeation of clean permeates through the membranes.

The surface charge of both of the membranes was measured as − 0.937 mV and − 5.25 mV for 4A-TPC@Crosslinked PAN and 4A-3P-TPC@Crosslinked PAN membranes, respectively, as shown in Figure S1.

### OSN performance of the membranes

**Figure 10:**
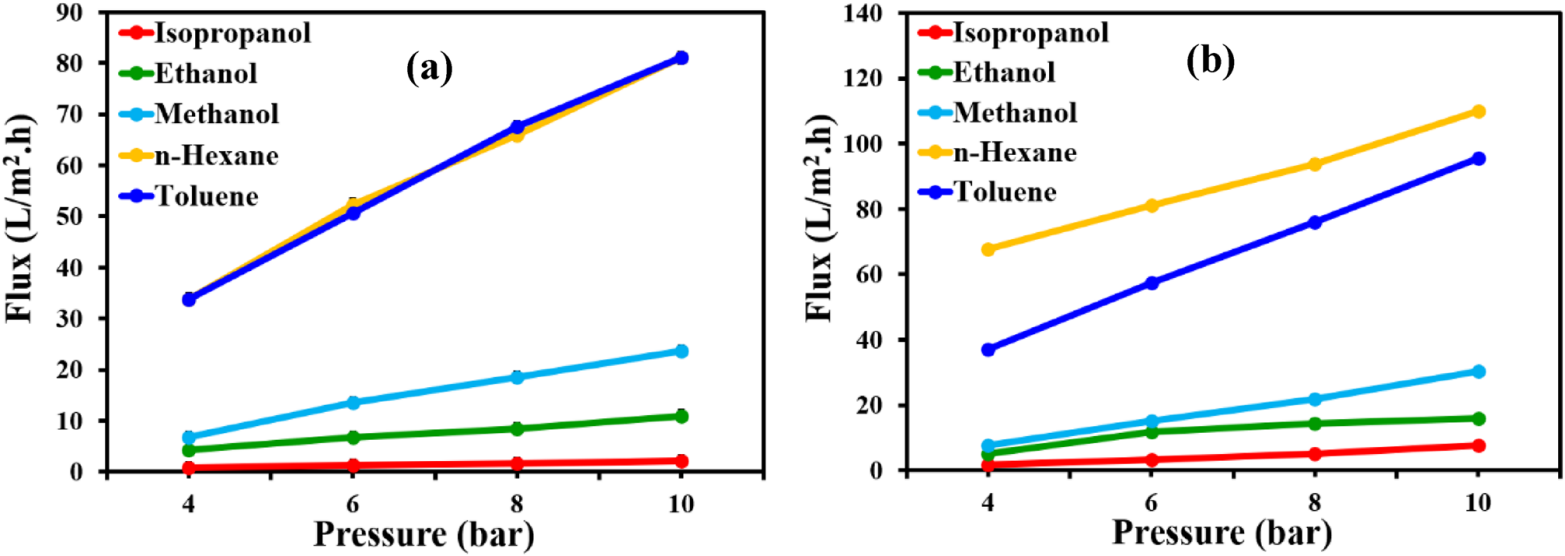
Variation of permeate flux as a function of applied feed pressure (4, 6, 8 and 10 bar) using (**a**) 4A-TPC@Crosslinked PAN and (**b**) 4A-3P-TPC@Crosslinked PAN membranes.

Following thorough characterization of the membranes, the OSN performance of the 4A-TPC@Crosslinked PAN and 4A-3P-TPC@Crosslinked PAN membranes was studied by using different polar and non-polar solvents including methanol, ethanol, isopropanol, *n*-hexane and toluene as feed in a dead end filtration cell. The effect of increasing transmembrane pressure on permeate flux was studied as given in Fig. 10. The flux was found to be dependent upon transmembrane pressure as the permeate flux of all of the tested solvents increased with increasing transmembrane pressure. The permeate flux of *n*-hexane and toluene was found to be the highest among the tested solvents. In the case of the 4A-TPC@Crosslinked PAN membrane, the permeate flux of n-hexane and toluene was raised from 33.8 LMH to 81.1 LMH as the pressure was increased from 4 to 10 bar (Fig. 10a). Similar findings were also found for the 4A-3P-TPC@Crosslinked PAN membrane. However, the permeate flux of all of the solvents was found to be higher compared to 4A-TPC@Crosslinked PAN membrane (Fig. 10b). The *n*-hexane and toluene showed a flux of 109.9 LMH and 95.5 LMH, respectively. This might be attributed to the existence of permissible channels for the passage of solvents through the 4A-3P-TPC@Crosslinked PAN membrane due to its flexibility for crosslinking during IP. In the case of polar solvents methanol showed the highest permeate flux.

**Figure 11:**
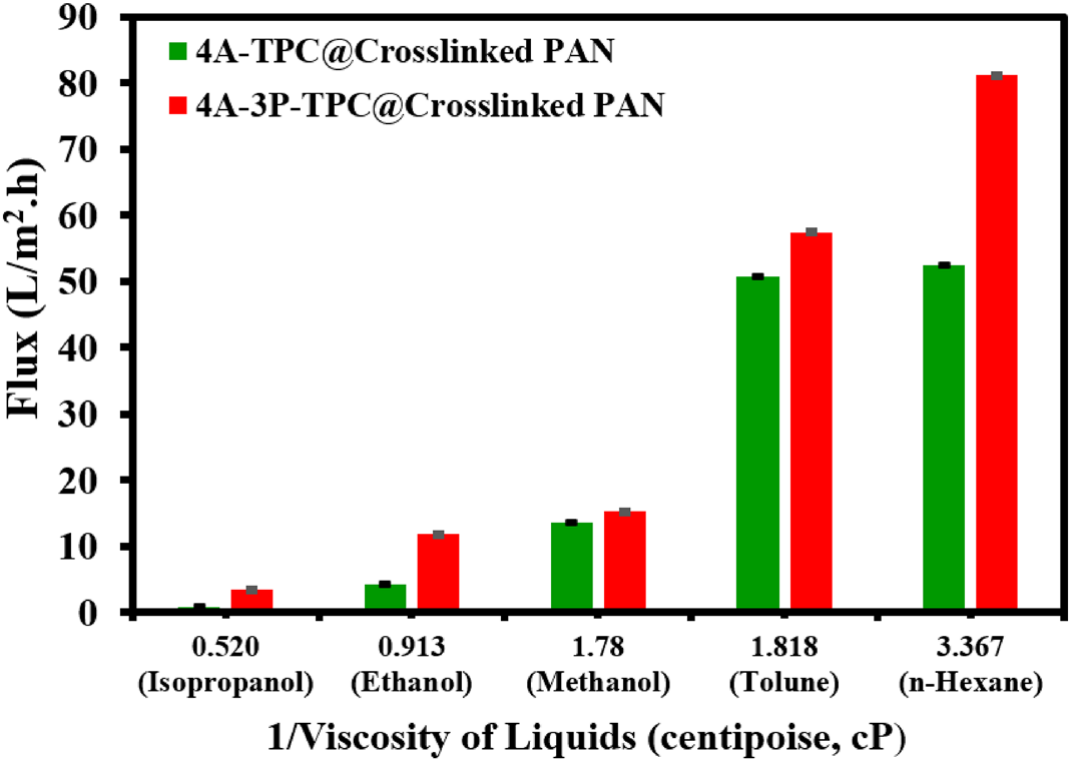
Effect of solvent viscosity on permeate flux of different solvents using 4A-TPC@Crosslinked PAN and 4A-3P-TPC@Crosslinked PAN membrane at a fixed applied pressure of 6 bar.

The variation in permeate flux can also be explained by considering another factor, which is the viscosity of the solvent. It has been observed that as the viscosity of the solvent increases, the permeate flux decreases in an inverse pattern. The inverse relationship between viscosity and permeate flux can be seen in Fig. 11. The *n*-hexane with the lowest viscosity (0.297 cP) showed the highest permeate flux, reaching 80 LMH, while isopropanol possessed the lowest permeate flux of 3 LMH and had the highest viscosity of 1.92 cP.

**Figure 12:**
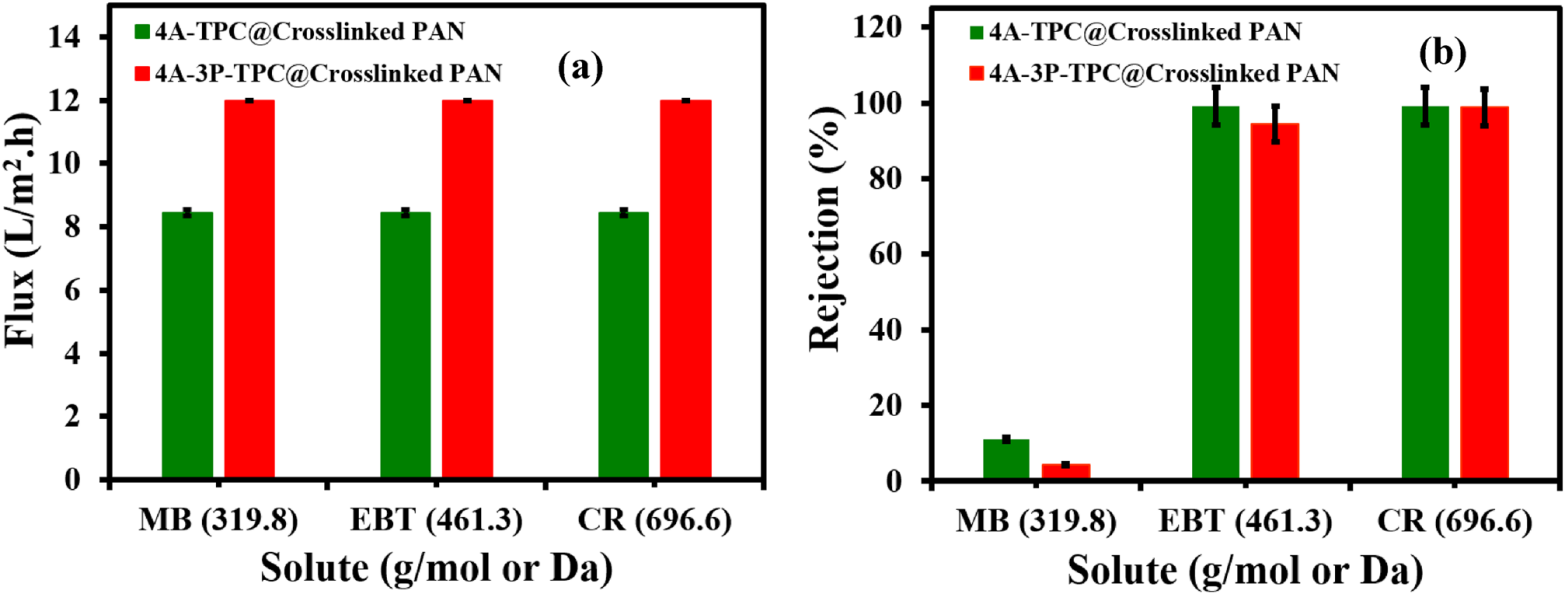
(**a**) Variation of permeate flux as a function of molecular weight of solutes (dyes) and (**b**) rejection performance of the 4A-TPC@Crosslinked PAN and (**b**) 4A-3P-TPC@Crosslinked PAN membrane for different dyes (solvent used = methanol; applied feed pressure = 4 bar).

The separation potential of OSN/SRNF membranes was also studied by using different dyes such as CR, EBT, and MB as model solutes. The solution of each dye was prepared by dissolving an appropriate amount of the dye in methanol at a concentration of 10 ppm. The feed of each dye was loaded in the dead-end cell and filtration was carried out at 4 bar. The flux of both membranes remained constant for all of the tested dyes (Fig. 12a,b). During the filtration experiments, the permeate flux of clean methanol was found to be 8.2 LMH for the 4A-TPC@Crosslinked PAN membrane while 12 LMH for 4A-3P-TPC@Crosslinked PAN membrane (Fig. 12a). The rejection performance of the membranes was analyzed by recording the absorption spectra of the permeates collected during filtration experiments and the rejection data is given in Fig. 12b. The CR showed the highest rejection reaching 99.1% for 4A-TPC@Crosslinked PAN membrane and 98.8% for 4A-3P-TPC@Crosslinked PAN membrane. In the case of EBT, the rejection stayed at 99.1% for 4A-TPC@Crosslinked PAN membrane while it was slightly reduced for 4A-3P-TPC@Crosslinked PAN membrane reaching 94.4%. Therefore, the 4A-3P-TPC@Crosslinked PAN membrane proved advantageous compared to 4A-TPC@Crosslinked PAN membrane as it has a comparatively higher permeate flux with comparable rejection of the solutes. Hence, a slight variation in the structure of the reacting monomers during IP alters the performance of the membrane during filtration experiments. The rejection of MB remained considerably lower compared to CR and EBT with 4.4% for 4A-3P-TPC@Crosslinked PAN membrane and 11.1% 4A-TPC@Crosslinked PAN membrane. The rejection of the dyes was also attempted with non-polar solvents but dyes were suspended in the solvent instead of dissolution (Supplementary Fig. S2).

**Figure 13:**
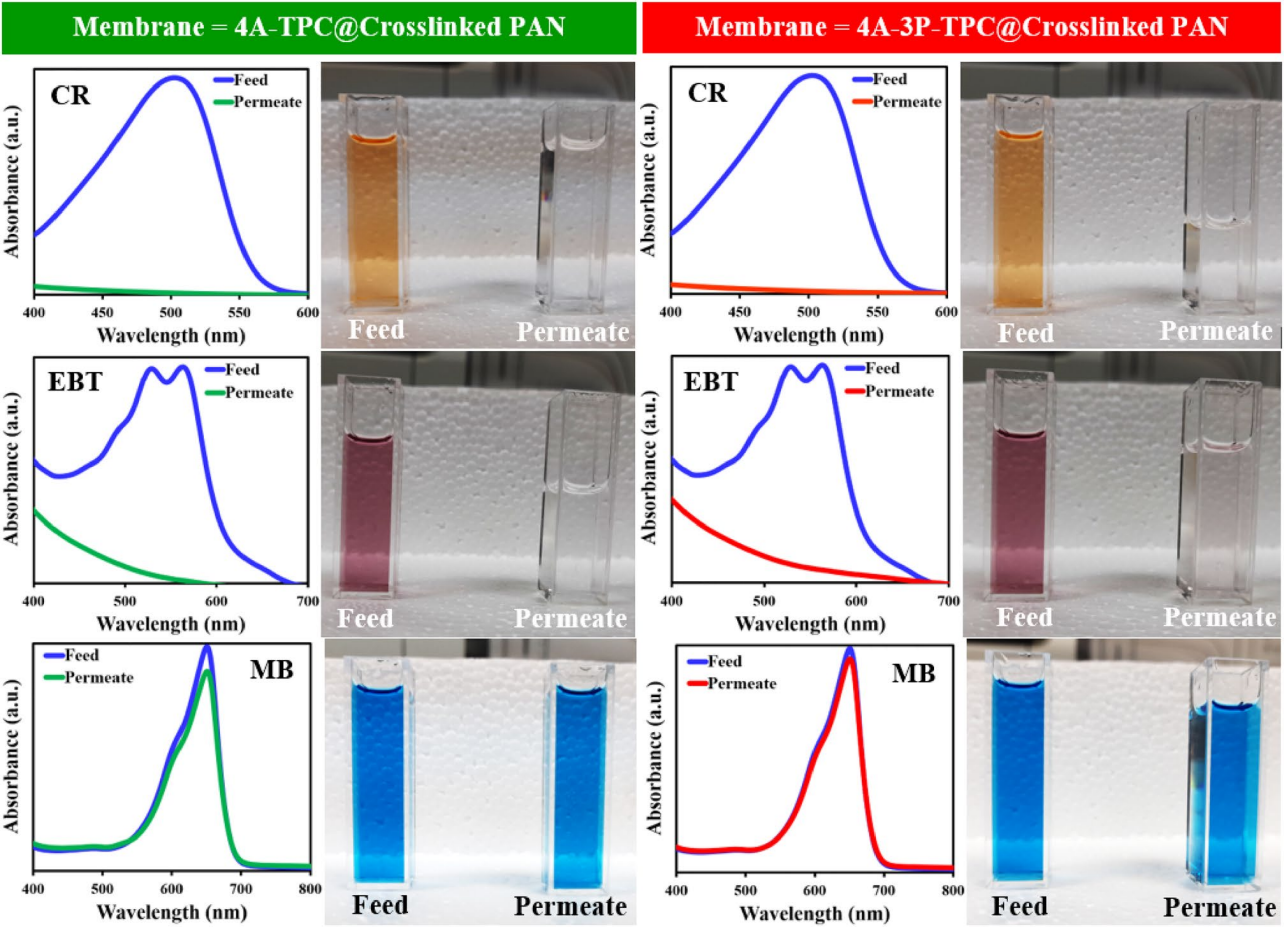
A comparison of the absorption spectra and coloration of feeds and permeates of CR, EBT and MB collected during filtration experiments (solvent used = methanol; Applied feed pressure = 4 bar).

Figure 13 shows the photographs along with the absorption spectra of the experimental samples of feeds and permeates of CR, EBT, and MB. It is clear from the following Fig. 13 for both membranes that absorption maximum of CR at 500 nm was completely flattened in the case of permeates which is also reflected by the colorful solution of CR in methanol and the colorless solution of permeates. Similarly, the absorption maximum of EBT at 550 nm was also flattened out in the case of permeates for both membranes. However, in the case of MB there was not a detectable difference in the absorption spectra and coloration of the feed and permeate samples.

The OSN/SRNF performances of the current membranes have been compared with those of similar membranes from the literature as given in Table 1. Compared to similar OSN membranes that have been reported in the literature, the 4A-TPC@Crosslinked PAN and 4A-3P-TPC@Crosslinked PAN membranes showed higher performance in terms of permeate flux of pure solvents and rejection of solutes, especially CR and EBT. However, the rejection of MB was found to be less compared to the membranes reported in the literature which might be due to the specific molecular weight cut off (MWCO) of the 4A-TPC@Crosslinked PAN and 4A-3P-TPC@Crosslinked PAN membranes.

**Table 1:** A comparison of PA(TEPA-TCL)@PSU/PETP membrane with the OSN/SRNF membranes from literature.

Membrane	Solvent	Flux (Lm^−2^ h^−1^)	Solute and molecular weight (g mol^−1^)	Rejection (%)	References
Molecularly porous hyper-cross-linked polyamide TFC-NF membrane	Methanol	4.5	CR (696.55)	> 94	^24^
			EBT (461.81)	> 72	
			MB (319.85)	> 90	
PA@PS/PET TFC-NF membrane	Methanol	3.69	CR (696.55)	> 95	^25^
			EBT (461.81)	> 96	
			MB (319.85)	> 67	
Polybenzimidazole (PBI) membranes					
PBI	Ethanol	~ 3.0	CR (696.55)	> 60	^26^
PBX	Ethanol	~ 2.0	CR (696.55)	> 75	
PCX	Ethanol	~ 2.5	CR (696.55)	> 70	
PMC	Ethanol	~ 3.2	CR (696.55)	> 70	
PBC	Ethanol	~ 1.8	CR (696.55)	> 65	
PCS	Ethanol	~ 1.9	CR (696.55)	> 70	
PMS	Ethanol	~ 2.0	CR (696.55)	> 70	
PA(TEPA-TCL)@PSU/PETP membrane	Methanol	6	CR (696.55)	99.91	^27^
			EBT (461.81)	96.92	
			MB (319.85)	87.85	
4A-TPC@Crosslinked PAN membrane	Methanol	8.2	CR (696.55)	99.1	This work
			EBT (461.81)	99.1	
			MB (319.85)	11.1	
4A-3P-TPC@Crosslinked PAN membrane	Methanol	12	CR (696.55)	98.8	This work
			EBT (461.81)	94.4	
			MB (319.85)	4.40	

## Conclusion

Interfacial polymerization has been successfully utilized for the fabrication of two thin film composite organic solvent nanofiltration/solvent resistant nanofiltration membranes. The structure of the polyamide active layers was tuned by slightly altering the chemistry of the reacting amines. Two different linear aliphatic amines, 4A with ethylene and 4A-3P with propylene, were used as aqueous amine solutions during IP on crosslinked PAN support. The resultant organic solvent nanofiltration/solvent resistant nanofiltration membranes 4A-TPC@Crosslinked PAN and 4A-3P-TPC@Crosslinked PAN membranes, were thoroughly characterized through scanning electron microscopy, water contact angle, energy disperse X-ray analysis, elemental mapping, attenuated total reflectance Fourier transform infrared spectrometry and atomic force microscopy. During organic solvent nanofiltration experiments, the 4A-TPC@Crosslinked PAN membrane showed an increase in permeate flux of n-hexane and toulene from 33.8 to 81.1 L m^−2^ h^−1^ as the pressure was increased from 4 to 10 bar. The 4A-3P-TPC@Crosslinked PAN membrane possessed n-hexane and toluene flux of 109.9 and 95.5 L m^−2^ h^−1^ respectively. The Congo red showed the highest rejection reaching 99.1% for 4A-TPC@Crosslinked PAN membrane and 98.8% for the 4A-3P-TPC@Crosslinked PAN membrane. In the case of Eriochrome Black T, the rejection stayed at 99.1% for 4A-TPC@Crosslinked PAN membrane while it was slightly reduced for 4A-3P-TPC@Crosslinked PAN membrane reaching 94.4%. Based on the rejection and flux data, the ethylene moeity between the two secondary amines led to a denser active layer compared to the propylene chain between the secondary amine groups of aliphatic amines.

## Supplementary Information

Supplementary Figures.

## Data Availability

The datasets used and/or analysed during the current study available from the corresponding author on reasonable request.
