# Supplementary material for: Fabrication of polyamide thin film composite membranes using aliphatic tetra-amines and terephthaloyl chloride crosslinker for organic solvent nanofiltration
Source: Sci Rep. 2023 Jul 20;13:11691. doi: 10.1038/s41598-023-38269-5 (PMC10359244; doi:10.1038/s41598-023-38269-5)
Supplement: Supplementary file 1 — Supplementary Figures. [file 41598_2023_38269_MOESM1_ESM.docx]

**Supplementary information**

**Fabrication of polyamide thin film composite membranes using aliphatic tetra-amines and terephthaloyl chloride crosslinker for organic solvent nanofiltration**

**Abdul Waheed^*1^, Umair Baig^*1^, Isam H. Aljundi^1,2^**

*^1^Interdisciplinary Research Center for Membranes and Water Security, King Fahd University of Petroleum and Minerals, Dhahran 31261, Saudi Arabia*

*^2^Chemical Engineering Department, King Fahd University of Petroleum & Minerals (KFUPM), Dhahran, 31261, Saudi Arabia*

***Corresponding Authors Contact Info:** abdul.waheed@kfupm.edu.sa (Dr. Abdul Waheed)**;** [umairbaig@kfupm.edu.sa](mailto:umairbaig@kfupm.edu.sa) (Dr. Umair Baig)


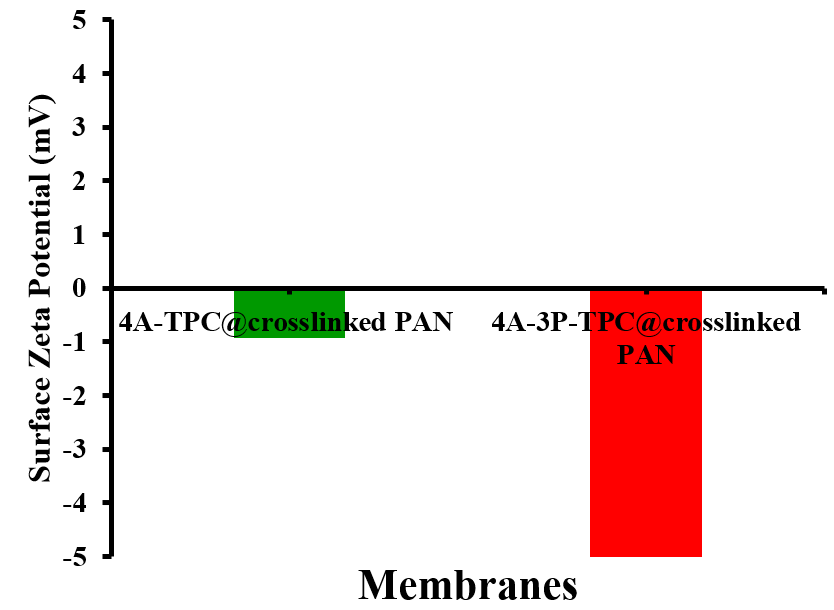


**Figure S1:** The Zeta-potential of the membranes prepared through IP.


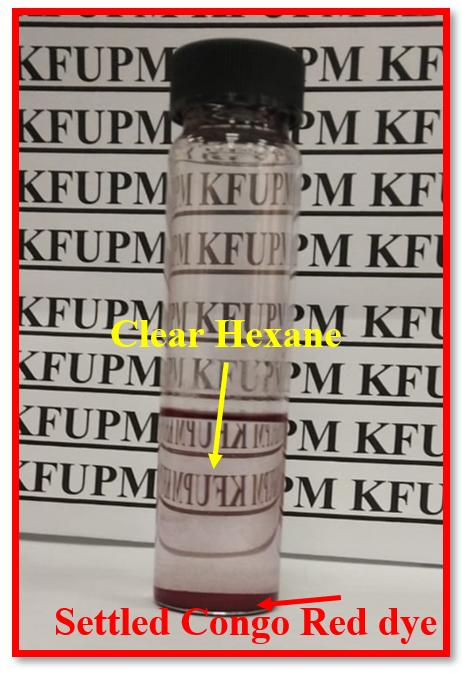


**Figure S2:** The solution of Congo red in n-hexane
